# β–Hydroxy β–Methylbutyrate Improves Dexamethasone-Induced Muscle Atrophy by Modulating the Muscle Degradation Pathway in SD Rat

**DOI:** 10.1371/journal.pone.0102947

**Published:** 2014-07-17

**Authors:** Kyung Kyun Noh, Ki Wung Chung, Yeon Ja Choi, Min Hi Park, Eun Ji Jang, Chan Hum Park, Changshin Yoon, Nam Deuk Kim, Mi Kyung Kim, Hae Young Chung

**Affiliations:** 1 Molecular Inflammation Research Center for Aging Intervention (MRCA), Department of Pharmacy, Pusan National University, Busan, Republic of Korea; 2 Longevity Life Science and Technology Institute, Pusan National University, Busan, Republic of Korea; McGill University, Canada

## Abstract

Skeletal muscle atrophy results from various conditions including high levels of glucocorticoids, and β–hydroxy β–methylbutyrate (HMB; a metabolite of leucine) is a potent therapeutical supplement used to treat various muscle disorders. Recent studies have demonstrated that HMB inhibits dexamethasone-induced atrophy in cultured myotubes, but its effect on dexamethasone-induced muscle atrophy has not been determined *in* *vivo*. In the present study, we investigated the effect of HMB on dexamethasone-induced muscle atrophy in rats. Treatment with dexamethasone weakened grip strengths and increased muscle damage as determined by increased serum creatine kinase levels and by histological analysis. Dexamethasone treatment also reduced both soleus and gastrocnemius muscle masses. However, HMB supplementation significantly prevented reductions in grip strengths, reduced muscle damage, and prevented muscle mass and protein concentration decrease in soleus muscle. Biochemical analysis demonstrated that dexamethasone markedly increased levels of MuRF1 protein, which causes the ubiquitination and degradation of MyHC. Indeed, dexamethasone treatment decreased MyHC protein expression and increased the ubiquitinated-MyHC to MyHC ratio. However, HMB supplementation caused the down-regulations of MuRF1 protein and of ubiquitinated-MyHC. Furthermore, additional experiments provided evidence that HMB supplementation inhibited the nuclear translocation of FOXO1 induced by dexamethasone, and showed increased MyoD expression in the nuclear fractions of soleus muscles. These findings suggest that HMB supplementation attenuates dexamethasone-induced muscle wasting by regulating FOXO1 transcription factor and subsequent MuRF1 expression. Accordingly, our results suggest that HMB supplementation could be used to prevent steroid myopathy.

## Introduction

Supplementation with the amino acid leucine and its metabolite a-ketoisocaproate (KIC) has been at the focus of investigations into skeletal muscle disorders for some 35 years, that is, since they were discovered to be potent anti-catabolic compounds [1]. β-Hydroxy β-methylbutyrate (HMB), another metabolite of leucine, has also been an interesting target for studies since Nissen et al. [2], [3] demonstrated its efficacy as a potent therapeutical supplement for the treatment of muscle disorders. Following studies showed that HMB might attenuate the muscle mass loss caused by AIDS [4], endotoxemia [5], [6], and aging [7], and that this effect is achieved by the inhibition of protein degradation and/or stimulated protein synthesis. In addition, recent studies have demonstrated that HMB supplementation in cachexia dampens skeletal muscle degradation and promotes protein synthesis [8], [9]. Furthermore, some authors have suggested that MAP kinase (Mitogen-activated protein kinase) and the PI3K (Phosphoinositide 3-kinase)/Akt signaling pathways are involved in these beneficial effects of HMB on skeletal muscle [6], [10], [11], [12]. However, the molecular mechanisms involved have not been determined and the effects of HMB not fully investigated.

Glucocorticoids are used as therapeutic agents due to their potent anti-inflammatory and immunosuppressive functions [13]. Despite of its advantage usage, high doses or the sustained usage of glucocorticoids by cortisol producing adrenal tumors (Cushing’s syndrome) or treatment with steroids for inflammatory conditions are associated with muscle wasting and weakness [14]. Dexamethasone-induced muscle atrophy is caused by catabolic conditions and the roles of glucocorticoids during muscle wasting are complex and reflect regulation at the molecular level of multiple mechanisms influencing both the synthesis and degradation of muscle proteins [15], [16]. The importance of the role played by glucocorticoids in the regulation of muscle atrophy is further demonstrated by various catabolic conditions, such as, sepsis and burn injury, that cause muscle wasting, at least in part, via glucocorticoid-dependent mechanisms [17]. Collectively, dexamethasone-induced muscle loss has significant biological meaning and provides an excellent model for investigating muscle atrophy.

The molecular mechanisms of glucocorticoid-induced muscle atrophy have been well studied. In particular, two muscle specific E3 ubiquitin ligases, namely muscle ring finger protein 1 (MuRF1) and atrogin-1/muscle atrophy F-box (MAFbx) have been found to play a key role in the process [15], [18]. It has been well demonstrated that MuRF1 and atrogin-1 ubiquitinate sarcomeric proteins, such as, myosin heavy chain (MyHC), which are then targeted for degradation through ubiquitin proteasome pathways [15]. Atrogin-1 also targets myogenic differentiastion1 (MyoD) and eukaryotic initiation factor 3 subunit 5 (eIF3-f), which play key roles in the control of protein synthesis [19]. Although the expressions and activities of MuRF1 and atrogin-1 are regulated by multiple mechanisms [18], studies suggest that members of the Forkhead box O (FOXO) family of transcription factors play an important role. In fact, MuRF1 and atrogin-1 are transcriptionally activated by FOXO1 and FOXO3 in skeletal muscle [20], [21], and FOXO1 plays a pivotal role in the expressional regulations of MuRF1 and atrogin-1 in various muscle atrophy-related conditions [20]. In the normal state, activation of the insulin like growth factor-1 (IGF-1)/PI3K/AKT pathway suppresses the expressions of MuRF1 and atrogin1 by phosphorylating and inactivating FOXO1 [21]. Mechanistically, muscle atrophy inducing factor induces the dephosphorylation, and thus, activates of FOXO1, which results in its nuclear translocation and the transcriptional activations of atrophy-inducing MuRF1 and atrogin-1 [22], [23]. Furthermore, the underlying mechanisms responsible for regulating FOXO1 expression and activity during muscle wasting, including glucocorticoid induced atrophy, have important clinical implications.

Recently, HMB was reported to influence the expressions of MuRF1 and MAFbx in glucocorticoid-induced muscle atrophy *in* *vitro* [24]. However, it is not clear whether HMB could prevent glucocorticoid-induced muscle atrophy *in* *vivo*. Thus, the aim of the present study was to investigate the effects of HMB supplementation on dexamethasone-induced skeletal muscle atrophy *in* *vivo*. In addition, we evaluated the molecular mechanisms involved in dexamethasone induced muscle atrophy and the inhibitory effects of HMB supplementation on these molecular events, in the hope that these evaluations would provide information regarding the therapeutic implications of HMB supplementation in a background of glucocorticoid-induced muscle atrophy.

## Methods and Materials

### Materials

All chemical reagents were obtained from Sigma-Aldrich (St. Louis, MO, USA), except where noted. HMB were obtained from Suppz Inc. (Fennimore, WI, USA). Antibodies against MyHC, MuRF1, MAFbx, FoxO1, MyoD, α-tubulin, TF-IIB, ubiquitin, anti-rabbit IgG-horseradish peroxidase-conjugated antibody, and anti-mouse IgG-horseradish peroxidase-conjugated antibody were obtained from Santa Cruz Biotechnology (Santa Cruz, CA, USA). Polyvinylidene difluoride (PVDF) membranes were obtained from Millipore (Bedford, MA, USA).

### Animal experiments

All animal studies were approved by the Institutional Animal Care Committee of Pusan National University and were performed in accordance with the guidelines for animal experimentation issued by Pusan National University. Six-week-old Sprague Dawley (SD) rats were obtained from Samtako (Osan, Korea). Rats were maintained under controlled environmental conditions under a 12 h/12 h light/dark cycle and were allowed *ad libitum* access to water and a standard laboratory diet. Animals were randomly divided into 5 groups of 6 animals after a 2-week acclimation period. Dexamethasone (600 µg/kg body mass) or normal saline were intraperitoneally (i.p.) injected once per day for five days. HMB (150 or 600 mg/kg/day) was orally administrated to designated groups. Leucine supplementation (600 mg/kg/day) group was used as positive control. During the experiments periods, forelimb grip strength was determined using a grip strength meter equipped with a T-shaped pull bar (Columbus Instruments, OH, USA). After the designated experiment schedule, rats were sacrificed by decapitation and soleus and gastrocnemius muscles were quickly removed. Muscle weights were immediately measured, and muscles were divided for biological analysis and histological examination.

### Measurements of serum glucose and insulin levels

Blood was collected and serum samples were separated by allowing blood to clot on ice. The serum so obtained was stored at −80°C for further analysis. Serum insulin was measured using commercially available kits (Shibayagi, Japan) and glucose levels were measured using commercially available kits (Shinyang Chemical, Seoul, South Korea), according to the manufacturer’s instructions.

### Assay for creatine kinase

Serum creatine kinase level was used as a marker of muscle damage. Level of creatine kinase was determined using an assay kit (SICDIA CPK; Shinyang chemical, Seoul), according to the manufacturer’s instructions. Absorbance was read immediately at 340 nm using a microplate reader (TECAN, Salzburg, Austria) and 5 min later. Creatine kinase activity was calculated by subtracting the initial reading from the second reading.

### Histological analysis

The medial portions of soleus muscles were fixed in 10% formalin solution for 24 h, routinely embedded in paraffin blocks, transversely sectioned (3 µm), and stained with hematoxylin and eosin. Muscle histologies were analyzed using an AE-31 light microscope (Motic, Hong Kong).

### Tissue homogenization

Whole soleus muscle was homogenized in homogenate buffer containing 50 mM HEPES (pH 7.4), 10 mM KCl, 2 mM MgCl2, 1 mM DTT, 0.1 mM EDTA, 0.1 mM PMSF, 20 mM β-glycerophosphate, 20 mM NaF, 2 mM Na_3_VO_4_, 1 µM pepstatin, 2 µM leupeptin and 5 µM aprotinin, and the homogenates obtained were placed on ice for 15 min. Nonidet P-40 (NP-40; 10%, 125 µl) solution was then added, mixed for 15 sec, and the mixture was then centrifuged at 14,000 *g* for 2 min. The supernatants obtained were used as cytosol fractions. Nuclear pellets were washed once, centrifuged, suspended in buffer containing 50 mM KCl, 300 mM NaCl, 0.1 mM PMSF, 10% (v/v) glycerol, 20 mM β-glycerophosphate, 20 mM NaF, 2 mM Na_3_VO_4_, 1 µM pepstatin, 2 µM leupeptin and 5 µM aprotinin, and kept on ice for 30 min. Mixtures were then centrifuged at 14,000 *g* for 10 min, and harvested supernatant were designated nuclear fractions. Protein concentrations were measured using the bicinchonic acid (BCA) method using bovine serum albumin (BSA) as a standard.

### Immunoblotting

Nuclear or cytosolic proteins (20∼100 µg of protein) were boiled for 5 min in gel-loading buffer (0.125 M Tris–HCl, pH 6.8, 4% SDS, 10% 2-mercaptoethanol, and 0.2% bromophenol blue) at a volume ratio of 1∶1. Samples containing the same amounts of proteins were then separated by sodium dodecyl sulfate–polyacrylamide gel electrophoresis in 8%∼15% acrylamide gels and transferred using a Bio-Rad western system (Bio-Rad, Hercules, CA, USA) to PVDF membranes, which were immediately placed in blocking buffer (5% non-fat milk) containing 10 mM Tris (pH 7.5), 100 mM NaCl, and 0.1% Tween 20. Membranes were then washed in TBS-Tween buffer for 30 min, incubated with specific primary antibodies (dilution 1∶250 to 1∶1000) at 4°C overnight, washed for 3×10-min in TBS-Tween buffer, and incubated with an horseradish peroxidase-conjugated anti-mouse antibody (Santa Cruz, 1∶10,000), an anti-rabbit antibody (Santa Cruz, 1∶10,000), or an anti-goat antibody (Santa Cruz, 1∶10,000) at 25°C for 1 h. The resulting immunoblots were visualized using Western Bright Peroxide solution (Advansta, CA, USA) and Davinch-chemi CAS-400 (Davinch-K, Seoul, Korea), according to the manufacturer’s instructions.

### Immunoprecipitation

Homogenized tissue proteins were subjected to immunoprecipitation (IP) in a buffer containing 40 mM Tris-HCl (pH 7.6), 120 mM NaCl, 20 mM β-glycerophosphate, 20 mM NaF, 2 mM sodium orthovanadate, 5 mM EDTA, 1 mM PMSF, 0.1% NP40 with leupeptin (2 µg/ml), aprotinin (1 µg/ml), and pepstatin A (1 µg/ml). An aliquot of protein extract (200 µg) was incubated with the required primary antibody for 4 h at 4°C, and again incubated overnight at 4°C with the appropriate protein A agarose or protein G agarose. After washing immunoprecipitates three times with IP buffer, pellets were dissolved in buffer containing 12.5 mM tris[hydroxymethyl] aminomethane, 4% sodium dodecylsulfate (SDS), 20% glycerol, 10% 2-mercaptoethanol, and 0.2% bromo-phenol blue (pH 6.8) at a vol. ratio of 1:1, and boiled for 5 min. After cooling, samples were spun-down briefly and supernatants were used in experiments.

### RT-PCR

Total RNA was isolated from tissues using TRIZOL (Invitrogen, Grand Island, NY, USA) and electrophoresed on a 1% agarose/MAE buffer gel (containing 50% formamide, 2.2 M formaldehyde, 1 mM 4-morpholinopropanesulfonic acid (MOPS), 0.4 M sodium acetate, 0.05 mM EDTA (pH 7.0)) to examine RNA degradation. cDNA was synthesized from 2 µg of isolated RNA. DEPC-treated water and 250 ng of random primer were added to RNA and incubated at 75°C for 5 min on ice for 5 min. Aliquots (2 µl) of a mixture of 0.1 M DTT, 4 µl of 5X buffer, 4 µl of 2.5 mM dNTP, 100 U of reverse transcriptase, and 16.5 U of RNase inhibitor were then added and incubated at 37°C for 2 h. The reaction was stopped by boiling at 100°C for 2 min, and cDNA was stored at −20°C until required. cDNA amplification was performed in a PCR master mix containing 1X PCR buffer (Perkin Elmer, Gaithersburg, MA, USA), 0.2 mM dNTP, 0.25 U of Taq polymerase (Perkin Elmer), and 50 ng of sense and anti-sense primers. The primers used were as follows: MuRF1, sense 5′-TTC ATC GAG GCC CTG ATC CT-3′ and anti-sense 5′-CTT GGC TTC CTT CCC CCT TT-3′; MyHC, sense 5′-TGC CAA GAC CGT GAG GAA TG-3′ and anti-sense 5′-AAT GCA TCA CAG CTC CCG TG-3′; and GAPDH, sense 5′-GGG TGA TGC TGG TGC TGA GTA TGT-3′ and anti-sense 5′-AAG AAT GGG AGT TGC TGT TGA AGT C-3′. PCR was conducted using 94°C for 30 sec denaturation, 54°C for 30 sec annealing, and 72°C for 1 min extension. Electrophoresis was performed in 1% agarose gel. After staining gels with ethidium bromide solution, they were observed under a UV transilluminator. GAPDH was used as internal control.

### Statistical Analysis

Data are expressed as means ± SEMs, and analyzed by using GraphPad Prism (version 5.0, GraphPad software, Inc.). Analysis of variance (ANOVA) was used to analyze intergroup differences. Differences among groups with a *P*<0.05 were considered significant by Dunnett’s multiple comparison tests when there were significant differences among groups by ANOVA.

## Results

### Effects of HMB supplementation on dexamethasone-induced atrophy, body weight, muscle wasting, and glucose homeostasis

**Figure 1:**
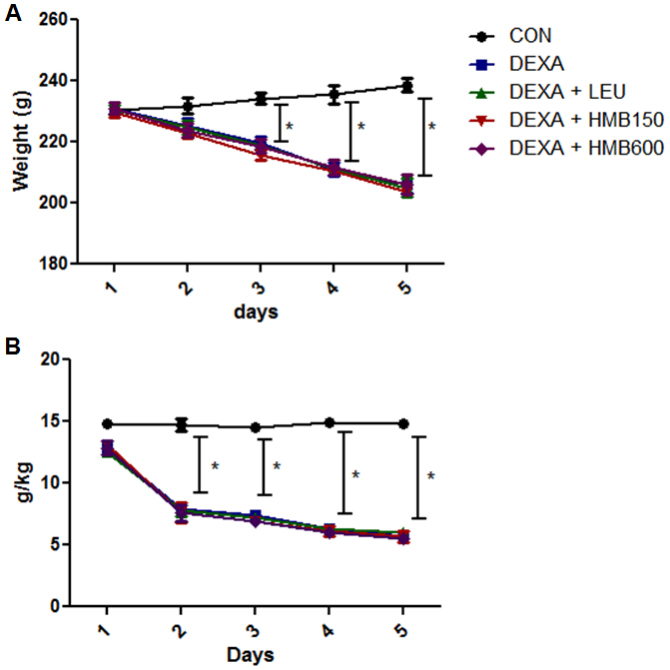
Changes in body weight and food intake during the experimental period. (A) Body weight changes in each group. *p<0.05; significant with respect to untreated controls. (B) Food intake changes in each group during the experimental period. *p<0.05; significant with respect to the untreated controls.

**Figure 2:**
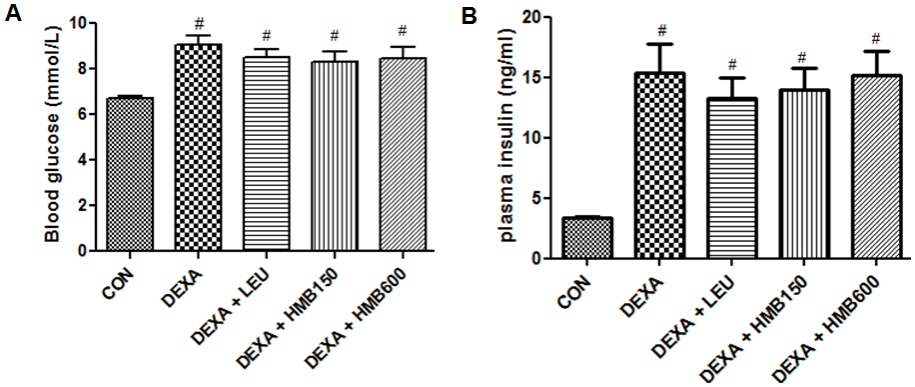
Serum glucose and insulin levels in the study groups. (A) Serum glucose levels after sacrifice. #p<0.05; significant difference between the treatment group and the controls group. (B) Serum insulin levels in the study groups after sacrifice. #p<0.05; significant difference between the treatment group and the control group.

Body weight changes and food intakes were measured during the 5-day experimental period. The dexamethasone-treated groups presented progressive weight loss throughout the study as compared with the control group (Table. 1, Fig. 1A). As was expected, dexamethasone treatment also caused a significant reduction in food intake (Fig. 1B). Supplementation of HMB or leucine did not prevent body weight loss or influence food intake (Table. 1). To investigate muscle loss after treatment, weights of soleus and gastrocnemius muscles were measured immediately after biopsy. Dexamethasone significantly decreased soleus and gastrocnemius weights (Table. 1), and supplementation of HMB or leucine did not prevent this muscle weight loss. To examine basal serum insulin level changes and glucose homeostasis, we checked serum insulin and glucose levels. Serum glucose levels were significantly increased by dexamethasone (Fig. 2A), but supplementation with leucine or HMB did not influence this dexamethasone-induced serum glucose increase. Serum insulin levels were markedly increased by dexamethasone (Fig. 2B), but neither supplement affected this dexamethasone-induced serum insulin increase. These results suggest that HMB supplementation does not influence dexamethasone-induced glucose or insulin increases.

**Table 1:** Body, gastrocnemius muscle, and soleus muscle weights of the experimental groups.

Variable	Group				
	CON	DEXA	DEXA-LEU	DEXA-HMB 150	DEXA-HMB 600
Basal BW (g)	230.5±5.4	231.4±4.6	233.1±4.4	229.2±3.6	231.7±3.9
Final BW (g)	238.7±5.6	205.6±7.1*	207.1±6.8*	204.4±7.2*	205.1±6.1*
Gastrocnemius muscle weight (g)	1.36±0.09	1.08±0.12*	1.09±0.08*	1.11±0.10*	1.10±0.09*
Soleus muscle weight (mg)	128.5±3.9	102.2±8.4*	105.6±6.2*	105.2±7.6*	109.3±5.9*

*p<0.05; significant difference between the control group and the treatment group.

BW, body weight; DEXA, Dexamethasone; LEU, Leucine 600 mg/kg/day Supplementation; HMB150, HMB 150 mg/kg/day supplementation; HMB 600, HMB 600 mg/kg/day supplementation.

### HMB supplementation mitigated dexamethasone-induced muscle atrophy by reducing muscle damage

**Figure 3:**
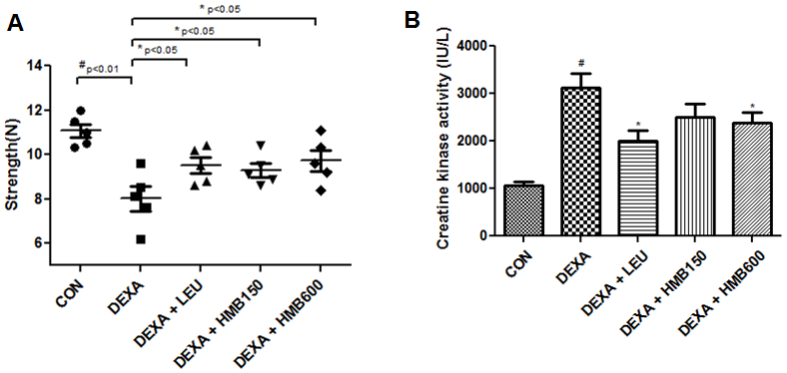
Forelimb grip strengths and serum creatine kinase levels. (A) Forelimb grip strengths in the study groups before sacrifice. Each dot represent the average of 5 tests per animal.^ #^p<0.01; significant difference between the dexamethasone group and the control group. *p<0.05; significant difference between the dexamethasone treated group and 3 other groups. (B) Serum creatine kinase levels in the study groups. Serum creatine kinase levels were measured after sacrifice.^ #^p<0.05; significant difference between the dexamethasone group and the control group. *p<0.05; significant difference between the dexamethasone group and the 3 other groups.

**Figure 4:**
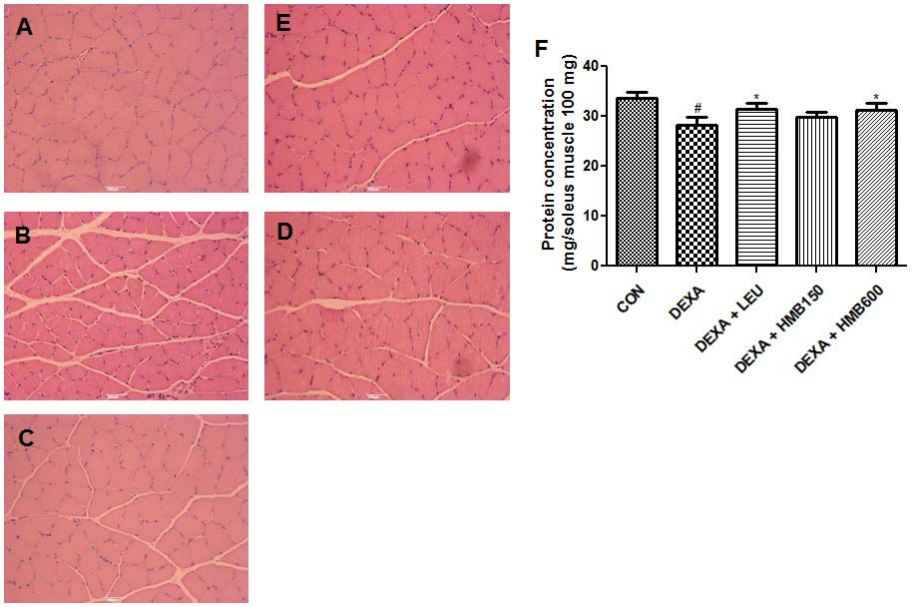
Histological analysis of soleus muscle cross sections. H&E stained sections of excised soleus muscle tissue in the control group (A), the dexamethasone treated group (B), the dexamethasone and leucine group (C), and the dexamethasone and HMB groups (D, E). Scale bar = 0.5 mm. (F) Protein concentration was measured in soleus muscles. ^#^p<0.05; significant difference between the dexamethasone group and the control group. *p<0.05; significant differences between the dexamethasone group and the other 3 groups.

In general, atrophy causes muscles to become weaker [16]. To determine muscle strength loss by dexamethasone, forelimb grip strength was measured with a grip strength meter. Dexamethasone treatment gradually reduced grip strength during the experimental periods, and significantly reduced grip strength on experimental day 5 versus the control groups (Fig. 3A). Supplementation of HMB or leucine effectively prevented this dexamethasone induced muscle weakness (Fig. 3A). Because the appearance of CK in blood serum is considered a surrogate marker of muscle damage, particularly for the diagnosis of myocardial infarction, muscular dystrophy, and cerebral diseases [25], we measured serum CK levels after 5 days of treatment. Treatment with dexamethasone significantly increased serum CK levels versus controls (Fig. 3B), and supplementation with HMB or leucine effectively reduced these dexamethasone-induced increases. We next examined muscle damage by histological staining of the medial portions of soleus muscles. Muscle fibers in the control group were in intimate contact in muscle bundles (Fig. 4A). However, dexamethasone caused severe damage to muscle bundles (Fig. 4B), which was ameliorated by supplementation with HMB or leucine (Fig. 4C∼4E). We also checked total protein concentrations in soleus muscles. Dexamethasone significantly reduced total protein concentrations in soleus muscles, whereas HMB or leucine reduced these reductions (Fig. 4F). These results suggest that HMB supplementation effectively prevented dexamethasone-induced muscle damage and muscle strength weakness.

### HMB supplementation reduced the ubiquitin-dependent proteolysis of MyHC

**Figure 5:**
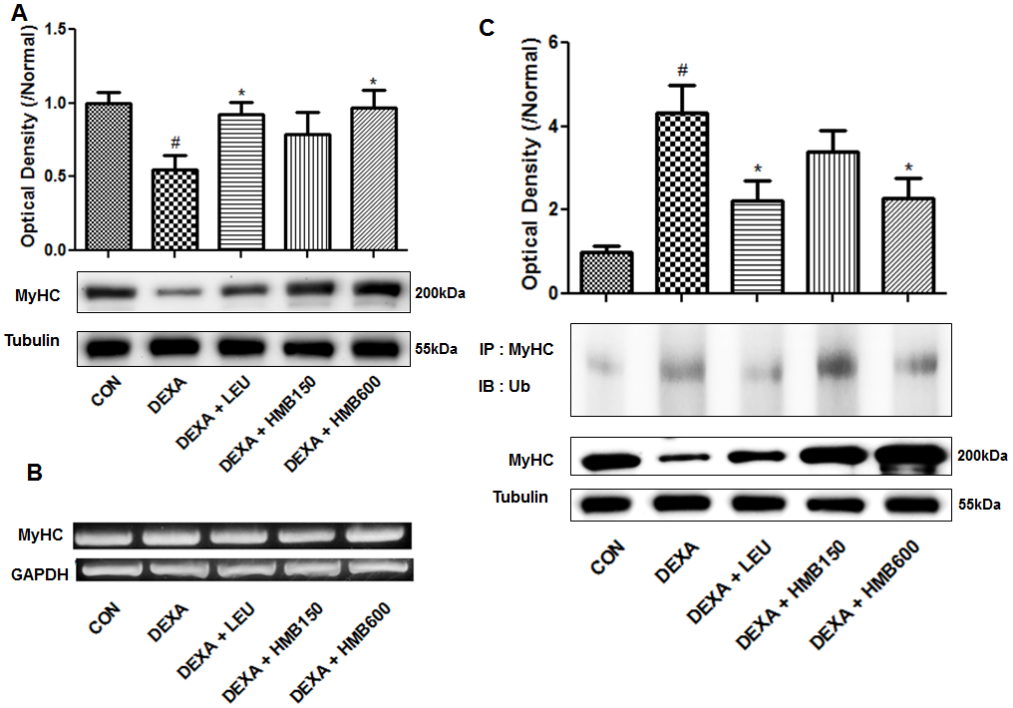
Effects of HMB supplementation on dexamethasone-induced MyHC degradation. (A) Representative western blot result of MyHC. Blots were quantified by densitometry and are expressed as relative to the control group. ^#^p<0.05; significant difference between the dexamethasone group and the control group. *p<0.05; significant difference between the dexamethasone group and the other four groups. (B) MyHC mRNA levels in soleus muscles for the study groups. One representative blot of each protein from three experiments that yielded similar results, is shown. (C) Detection of ubiquitinated-MyHC by immunoprecipitation. Representative western blot result of ubiquitinated-MyHC and MyHC in each study group. Ratios of ubiquitinated-MyHC to MyHC were calculated by densitometry and are expressed relative to control group values. ^#^p<0.05; significant difference between the dexamethasone group and the control group. *p<0.05; significant differences between the dexamethasone group and the other four groups.

Since dexamethasone is known to deplete myosin heavy chain (MyHC), which plays an important role in muscle contraction [16], we measured MyHC protein levels in soleus muscle. Dexamethasone markedly reduced MyHC protein levels (Fig. 4A), and supplementation with HMB or leucine effectively inhibited this reduction (Fig. 5A). We further investigated whether reductions in MyHC levels were caused by increased protein degradation or decreased protein synthesis. MyHC mRNA levels were not affected by dexamethasone treatment or supplementation (Fig. 5B), indicating that the synthesis of MyHC was not affected by treatment or supplementation. Since muscle degradation is largely due to the ubiquitination of MyHC, which is catalyzed by E3 ligase [16], we next analyzed its ubiquitination. Dexamethasone significantly increased the ratio of ubiquitinized-MyHC to total MyHC (Fig. 5C), and this increase was markedly reduced by supplementation with leucine or HMB (Fig. 4C). These results show that HMB supplementation inhibits dexamethasone induced muscle loss by reducing the ubiquitination of MyHC.

### HMB supplementation affected dexamethasone induced MuRF1 expression by modulating FOXO1

**Figure 6:**
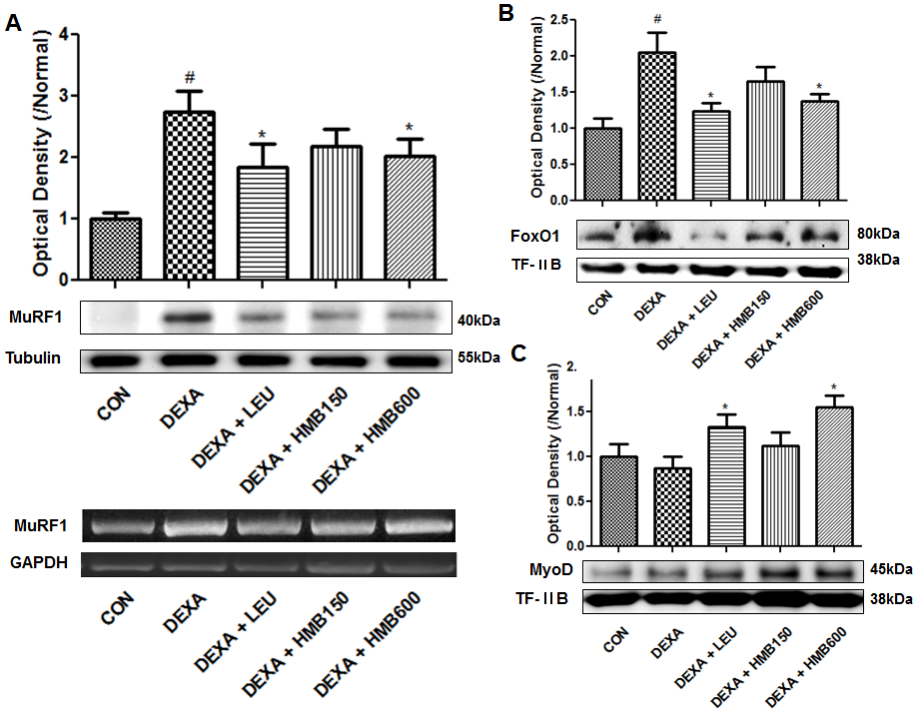
Effects of HMB supplementation on dexamethasone-induced MuRF1 and MyoD protein levels and on and in nuclear translocation of FOXO1. (A) Representative western blot of MuRF1 in each study group. Blots were quantified by densitometry and values are expressed versus the control group. ^#^p<0.05; significant difference between the dexamethasone group and the control group. *p<0.05; significant difference between the dexamethasone group and the other four groups. (B) Representative western blot result of nuclear FOXO1 in the study groups. The blots were quantified by densitometry and values are expressed relative to control group values. ^#^p<0.05; significant difference between the dexamethasone group and the control group. *p<0.05; significant difference between the dexamethasone group and the other four groups. (C) Representative western blot result of nuclear MyoD in each study group. The blots were quantified by densitometry and values are expressed relative to control values. ^#^p<0.05; significant difference between the dexamethasone group and the control group. *p<0.05; significant difference between the dexamethasone group and the other four groups.

We next examined the protein level of MuRF1 in soleus muscle, because it is a muscle specific E3 ligase responsible for the degradation of MyHC [16]. As was expected, dexamethasone markedly increased the protein and mRNA levels of MuRF1 (Fig. 6A), and supplementation with leucine or HMB significantly reduced these increases (Fig. 6A). However, protein and mRNA levels of atrogin-1 (another E3 ligase involved in muscle degradation) was unaffected by dexamethasone (data not shown). We also examined the nuclear translocation of the transcription factor FOXO1, which induces the expression of MuRF1. Dexamethasone was found to induce FOXO1 translocation (Fig. 6B), and the supplementation of HMB or leucine reduced this effect. We also examined the expression of MyoD, which is involved in muscle differentiation and repair (26). We found that whereas dexamethasone did not affect MyoD levels in nuclear fractions, HMB supplementation significantly increased these levels (Fig. 6C). These results suggest that HMB supplementation reduces MuRF1 expression by inhibiting the nuclear translocation of FOXO1 and by increasing nuclear MyoD expression.

## Discussion

Skeletal muscle atrophy is caused by various conditions, such as, sepsis, cancer, renal failure, glucocorticoid excess, denervation, muscle disuse, and the aging process [14], [27], and reduces treatment options, compromises quality of life, and increases morbidity and mortality [28], [29]. High glucocorticoid levels are associated with muscle wasting and weakness, as is observed in patients with Cushing’s syndrome and patients treated with corticosteroids for asthma and rheumatoid arthritis [14], [30], [31]. It has been shown that imbalance between rates of protein synthesis and breakdown contribute to muscle atrophy. In previous studies, ubiquitin dependent proteolysis was found to importantly contribute to protein breakdown associated with muscle atrophy [17], [32], [33]. Accordingly, interventions targeting protein breakdown are of interest, and several agents have been discovered and developed for the treatment of muscle atrophy [34], [35].

HMB is a metabolite of leucine, which is produced in the human body. In fact, HMB is sold as a supplement based on premise that improved protein synthesis in skeletal muscles [1]. Previous studies have demonstrated the protective effects of HMB on the catabolic states induced by cancer cachexia, AIDS, and muscular dystrophy [2]–[9]. These studies commonly demonstrated that HMB attenuates muscle wasting by inhibiting protein breakdown and stimulating protein synthesis. However, although many studies have demonstrated the protective effects of HMB on various catabolic conditions, these effects are not universal, for example, HMB did not prevent muscle wasting in patients with rheumatoid arthritis [36]. In another study, it was demonstrated that only protein degradation was improved by HMB and that it had no effect on protein synthesis [5], indicating the importance of determining the effects of HMB on muscle protein synthesis and degradation under different conditions. In a previous study, the mechanism responsible for the effects of HMB on dexamethasone induced muscle atrophy was studied *in* *vitro* [24]. Accordingly, we investigated the *in* *vivo* effects of HMB on dexamethasone-induced muscle atrophy.

In several studies that used glucocorticoids to induce catabolic states, physiological muscle changes were observed after dexamethasone treatment [16], [27]. For example, dexamethasone significantly induced muscle wasting and changed the balance of protein synthesis and degradation [16], [17], [21]. Our findings regarding the effects of dexamethasone concur with those of previous studies. In the present study, HMB was not found to reduce the body weight decreases induced by dexamethasone. However, HMB supplementation did significantly inhibit dexamethasone-induced reductions in muscle strength. To investigate the effect of dexamethasone muscle damage, we measured serum CK levels (a marker of muscle breakdown [25]) and examined excised muscles histologically. We found that HMB supplementation markedly reduced dexamethasone-induced serum CK level increases and alleviated muscle bundle damage. On the other hand, HMB supplementation had little effect on muscle weight reductions induced by dexamethasone, despite the finding that protein concentration reductions in soleus muscle by dexamethasone were inhibited by HMB supplementation. This discordance between the effects of HEM on dexamethasone-induced changes in muscle weight and muscle integrity requires further study. Nonetheless, in one study, it was shown that some markers of muscle atrophy can change without commensurate changes in body weight or muscle weight [37].

In catabolic states, PI3K/Akt signaling is not enough to inhibit FoxO factors [20], [38], [39]. The administration of dexamethasone is known to influence this signaling and to induce muscle protein degradation and synthesis imbalance [20], [21]. The nuclear translocation of FoxO results in the expressions of MuRF1 and MAFbx, which cause the ubiquitination and degradation of MyHC [16], [21], and thus, we expected that these signalings would be affected by HMB. Western blot analysis showed protein levels of MyHC were down-regulated by dexamethasone and that HMB inhibited the degradation of MyHC. However, the mRNA levels of MyHC remained the same in all groups, indicating that MyHC protein levels were regulated by protein degradation rather than by transcription. Since MyHC is degraded by a proteasome-dependent pathway [16], we checked the ubiquitination of MyHC, and found that the dexamethasone-induced ubiquitination of MyHC was markedly reduced by HMB supplementation. MuRF1 is known to catalyze the ubiquitination and degradation of MyHC [16], we next examined the MuRF1 expression in soleus muscle. MuRF1 expression was induced by dexamethasone but suppressed by HMB at the mRNA and protein levels. We then examined the transcriptional activity of FoxO1 by analyzing its levels in nuclear fractions. Levels of FoxO1 in these fractions were increased by dexamethasone and these increases were inhibited by HMB, which is consistent with our observations of MuRF1 expression. These findings suggest that HMB inhibited the ubiquitin-dependent proteolysis of MyHC by inhibiting FoxO1 translocation, and thus, modulating the expression of MuRF1. In particular, the observed effects of HMB on dexamethasone-induced muscle atrophy in the present study agree with observations made in a previous *in* *vitro* study [24]. Somewhat surprisingly although MAFbx appeared to have a role in muscle atrophy in previous studies [18], [26], in the present study, the mRNA and protein levels of MAFbx were unaffected by HMB (data not shown), presumably because MAFbx is not directly regulated by glucocorticoid receptor, whereas MuRF1 is directly regulated by glucocorticoid receptor and FoxO [21].

The effects of HMB on muscle regeneration were also investigated, because it is known to influence muscle repair mechanisms [8], [9]. MyoD plays an important role during early the differentiation and regeneration of muscle [19], [26], [40]. To examine MyoD transactivation, nuclear levels of MyoD were analyzed by Western blot analysis. Dexamethasone did not affect nuclear levels of MyoD, but HMB increased its nuclear levels. Further studies are needed to investigate the effects of HMB on the transactivation of MyoD and muscle regeneration. However, our data suggest that HMB supplementation could influence muscle regeneration.

**Figure 7:**
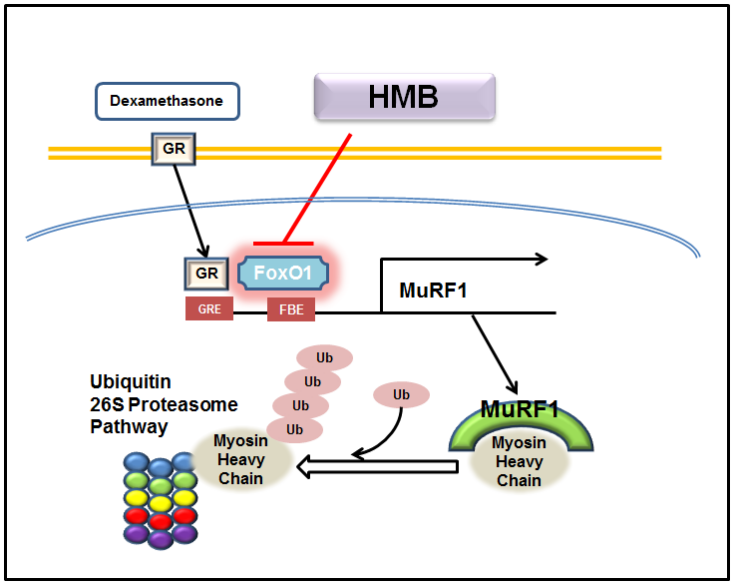
Proposed mechanism for the effect of HMB on dexamethasone-induced muscle atrophy.

The present study was limited by the fact that experiments were focused on physiological changes and on the expressions of well-known muscle atrophy-related proteins, and not on the mechanism responsible for the effects of HMB supplementation on dexamethasone-induced muscle atrophy. Nevertheless, we found that HMB supplementation decreased the nuclear translocation of FOXO1, which indicates that further studies are needed to demonstrate whether PI3K/Akt signals or other signals associated with FOXO are associated with the effects of HMB. Furthermore, because HMB supplementation had no effect on serum insulin, which importantly signals the induction of PI3K/Akt, we can only speculate that HMB influences PI3K/Akt signaling directly. However, our findings do show that HMB supplementation markedly reduced dexamethasone-induced muscle atrophy by down-regulating MuRF1 by inhibiting FoxO1 translocation, and that this inhibits MyHC degradation. In addition, our *in* *vivo* data show that HMB protects against glucocorticoid-induced muscle atrophy and suggest that HMB supplementation be considered a potential treatment for muscle atrophy (Figure 7).
